# Trienone analogs of curcuminoids induce fetal hemoglobin synthesis via demethylation at ^G^γ-globin gene promoter

**DOI:** 10.1038/s41598-021-87738-2

**Published:** 2021-04-20

**Authors:** Khanita Nuamsee, Thipphawan Chuprajob, Wachirachai Pabuprapap, Pornrutsami Jintaridth, Thongperm Munkongdee, Phatchariya Phannasil, Jim Vadolas, Pornthip Chaichompoo, Apichart Suksamrarn, Saovaros Svasti

**Affiliations:** 1 Graduate Program in Molecular Medicine, Faculty of Science, Mahidol University, Bangkok, Thailand; 2 Thalassemia Research Center, Institute of Molecular Biosciences, Mahidol University, Nakhon Pathom, Thailand; 3 Department of Pathobiology, Faculty of Science, Mahidol University, Bangkok, Thailand; 4 Department of Chemistry and Center of Excellence for Innovation in Chemistry, Faculty of Science, Ramkhamhaeng University, Bangkok, Thailand; 5 Department of Chemistry, Faculty of Science, Siam University, Bangkok, Thailand; 6 Department of Tropical Nutrition and Food Science, Faculty of Tropical Medicine, Mahidol University, Bangkok, Thailand; 7 Centre for Cancer Research, Hudson Institute of Medical Research, Melbourne, Australia; 8 Department of Molecular and Translational Science, Monash University, Melbourne, Australia; 9 Department of Biochemistry, Faculty of Science, Mahidol University, Bangkok, Thailand

**Keywords:** Drug discovery and development, Molecular medicine

## Abstract

The reactivation of γ-globin chain synthesis to combine with excess free α-globin chains and form fetal hemoglobin (HbF) is an important alternative treatment for β-thalassemia. We had reported HbF induction property of natural curcuminoids, curcumin (Cur), demethoxycurcumin (DMC) and *bis*-demethoxycurcumin (BDMC), in erythroid progenitors. Herein, the HbF induction property of trienone analogs of the three curcuminoids in erythroleukemic K562 cell lines and primary human erythroid progenitor cells from β-thalassemia/HbE patients was examined. All three trienone analogs could induce HbF synthesis. The most potent HbF inducer in K562 cells was trienone analog of BDMC (T-BDMC) with 2.4 ± 0.2 fold increase. In addition, DNA methylation at CpG − 53, − 50 and + 6 of ^G^γ-globin gene promoter in K562 cells treated with the compounds including T-BDMC (9.3 ± 1.7%, 7.3 ± 1.7% and 5.3 ± 0.5%, respectively) was significantly lower than those obtained from the control cells (30.7 ± 3.8%, 25.0 ± 2.9% and 7.7 ± 0.9%, respectively *P* < 0.05). The trienone compounds also significantly induced HbF synthesis in β-thalassemia/HbE erythroid progenitor cells with significantly reduction in DNA methylation at CpG + 6 of ^G^γ-globin gene promoter. These results suggested that the curcuminoids and their three trienone analogs induced HbF synthesis by decreased DNA methylation at ^G^γ-globin promoter region, without effect on ^A^γ-globin promoter region.

## Introduction

β-Thalassemia, one of the most common genetic disorders, is caused by the defect in β-globin gene leading to the reduced or absent synthesis of β-globin chain. The imbalanced globin chain synthesis results in excess unbound α-globins and pathologies in β-thalassemia patients, including chronic anemia, iron overload, hepatosplenomegaly, cardiac dysfunction and heart failure^1^. The current therapy for severe anemia in β-thalassemia patients is blood transfusion. However, the long-term blood transfusion causes a high rate of allo-immunization and iron overload. The only curative treatment is stem cell transplantation which requires compatibility of human leukocyte antigen (HLA)-match donor and long-term use of immunosuppressive drugs^1^. Due to these limitations, alternative treatment for β-thalassemia is necessary. The stimulation of γ-globin chain synthesis reduces the excess unbound α-globin as γ-globin chains assemble with the excess unbound α-globin chains to form HbF, consequently decreasing clinical severity of β-thalassemia^1^. Several HbF inducers have been evaluated in clinical trials, such as 5-azacytidine (5-Aza)^2^, decitabine (5-aza-2′-deoxycytidine)^3^, hydroxyurea (HU)^4^, butyrate and short chain fatty acids (SCFAs)^5, 6^ with modest response in β-thalassemia and sickle cell anemia patients. However, HU, the only FDA-approved HbF-inducing agent, is not an ideal drug due to drug adverse effects. Moreover, it also exhibits variable responders with an approximately 20–50% partial responders and non-responders in transfusion dependence β-thalassemia patients^7, 8^. Therefore, new agents that can induce HbF with less or no adverse effects are highly needed.

**Figure 1:**
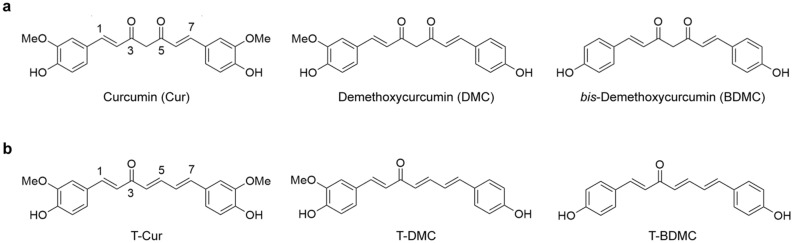
Structures of curcuminoids and trienone analogs of curcuminoids. (**a**) The structures of curcuminoids (Cur, DMC and BDMC) consist of two aromatic rings containing phenolic methoxy and/or hydroxy groups connected by an α,β-unsaturated β-diketo C_7_ chain. (**b**) The structures of trienone analogs of curcuminoids (T-Cur, T-DMC and T-BDMC) consist of two aromatic rings containing phenolic methoxy and/or hydroxy groups and the two aromatic rings are connected by a 1*E*,4*E*,6*E*-heptatrien-3-one C_7_ chain.

Induction of γ-globin mRNA expression and HbF synthesis in human erythroid progenitors by curcuminoids have been reported^9^. Curcuminoids consist of three notable compounds, a major constituent, curcumin (Cur), (1*E*,6*E*)-1,7-*bis*-(4-hydroxy-3-methoxyphenyl)-1,6-heptadien-3,5-dione, and two minor related analogs, demethoxycurcumin (DMC), (1*E*,6*E*)-1-(4-hydroxy-3-methoxyphenyl)-7-(4-hydroxyphenyl)-1,6-heptadien-3,5-dion, and *bis*-demethoxycurcumin (BDMC), (1*E*,6*E*)-1,7-*bis-*(4-hydroxyphenyl)-1,6-heptadien-3,5-dione (Fig. 1a). Trienone analogs of curcuminoids were recently found in natural and/or as synthetic analogs including (1*E*,4*E*,6*E*)-1,7-*bis*-(4-hydroxy-3-methoxyphenyl)-1,4,6-heptatrien-3-one (T-Cur), (1*E*,4*E*,6*E*)-1-(4-hydroxy-3-methoxyphenyl)-7-(4-hydroxyphenyl)-1,4,6-heptatrien-3-one (T-DMC) and (1*E*,4*E*,6*E*)-1,7-*bis*-(4-hydroxyphenyl)-1,4,6-heptatrien-3-one (T-BDMC), analogs of Cur, DMC and BDMC, respectively (Fig. 1b)^10^. These trienone compounds might also induce HbF synthesis.

DNA methylation is one of the epigenetic mechanisms that has been reported to control γ-globin gene activation and silencing during developmental stages^11^. Cytidine analogs, 5-Aza and decitabine, DNA methyltransferase I (DNMT1) inhibitors, have been reported to induce HbF synthesis in β-hemoglobinopathies^2, 3^. However, limitation of these compounds was concerned over carcinogenic potential and rapid degradation by the pyrimidine metabolism enzyme, cytidine deaminase^12, 13^. Cur has been reported to modulate DNA methylation by inhibit DNMT1 expression and its activity^14, 15^. In this study, effects of the curcuminoids and their trienone analogs on HbF synthesis and DNA methylation at both ^G^γ- and ^A^γ-globin gene promoters in K562 cells and primary human erythroid progenitors from β-thalassemia/HbE patients were examined. We found that HbF synthesis was induced by the trienone compounds and decreased DNA methylation level at ^G^γ-globin gene promoter was observed.

## Results

### Trienone analogs of curcuminoids induce HbF synthesis in K562 cells

**Figure 2:**
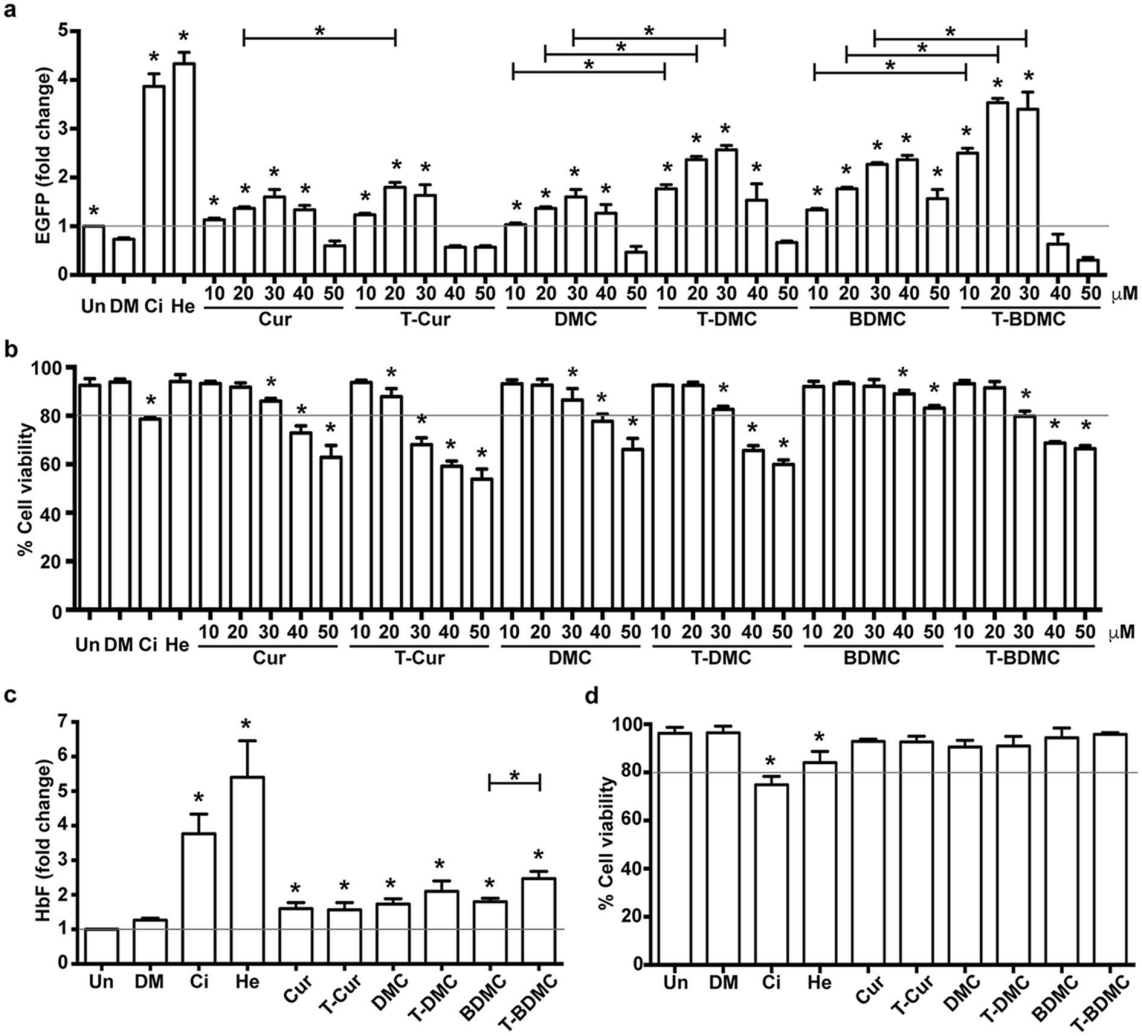
Curcuminoids and their trienone analogs induce HbF synthesis in K562 cells. K562::Δ^G^γ-^A^γ EGFP reporter cells and native K562 cells were treated with curcuminoids and their trienone analogs for 5 days. (**a**) EGFP expression and (**b**) cell viability of K562::Δ^G^γ-^A^γ EGFP reporter cells were analyzed by flow cytometry. Increased EGFP mean fluorescent intensity in a dose-dependent manner in treated cells compared with DMSO treated cells was observed. The concentration that yielding highest EGFP expression induction activity with more than 80% cell viability was selected for examined in native K562 cells. (**c**) HbF synthesis and (**d**) cell viability of native K562 cells were determined by flow cytometry. The results showed increased HbF synthesis in the compounds treated cells compared with the DMSO treated cells. Cisplatin (20 μM) and hemin (50 μM) were used as positive control. The fold changes in EGFP expression and HbF synthesis were compared with the untreated cells and showed as mean ± SD of three independent experiments. *, statistical significance when compared to DMSO treated cells or between groups at *P* < 0.05. Un, untreated; DM, DMSO; Ci, cisplatin; He, hemin.

The HbF inducing property of trienone analogs of curcuminoids was first examined in K562:Δ^G^γ-^A^γ EGFP reporter cells, a promoter-EGFP reporter, harboring enhance green fluorescent protein (EGFP) under control of the ^G^γ-globin promoter^16^. The cells were treated with curcuminoids (Cur, DMC and BDMC) or their trienone analogs (T-Cur, T-DMC and T-BDMC) (at 10, 20, 30, 40 and 50 μM). The curcuminoids and trienone analogs enhanced EGFP expression as a dose-dependent response manner (Fig. 2a). Among curcuminoids, BDMC showed the highest induction of EGFP expression, 2.3 ± 0.1 folds, compared to the DMSO treated cells (0.7 ± 0.1 folds) (*P* < 0.05) (Fig. 2a). In addition, its trienone analogs, T-BDMC, was the most effective trienone analogs inducing the 3.5 ± 0.2 folds EGFP expression compared to DMSO treated cells (*P* < 0.05) (Fig. 2a). The T-BDMC showed higher induction of EGFP expression than BDMC (*P* < 0.05). Cell viability of the cells treated with BDMC, 92.1 ± 2.9%, and T-BDMC, 91.6 ± 2.6%, was higher than cells treated with 20 μM cisplatin, 76.8 ± 1.8% (Fig. 2b).

Although, up-regulation of EGFP expression in K562::Δ ^G^γ-^A^γ EGFP reporter cells was promising, the EGFP coding sequence under the control of γ-globin promoter may not entirely represent the endogenous γ-globin gene expression of K562 cells. Therefore, HbF inducing property of these effective compounds in native K562 cells was quantified by flow cytometry using FITC conjugated monoclonal antibody against human fetal hemoglobin. Consistent with results in the K562::Δ^G^γ–^A^γ EGFP reporter cells, HbF synthesis in the K562 cells treated with T-BDMC was higher than cells treated with BDMC for 2.4 ± 0.2 and 1.8 ± 0.1 folds, respectively (*P* < 0.05) (Fig. 2c). Furthermore, cell viability of K562 cells treated with all six compounds at the most effective concentration was higher than 90% (Fig. 2d).

### Trienone analogs of curcuminoids decreased DNA methylation at γ-globin promoter in K562 cells

**Figure 3:**
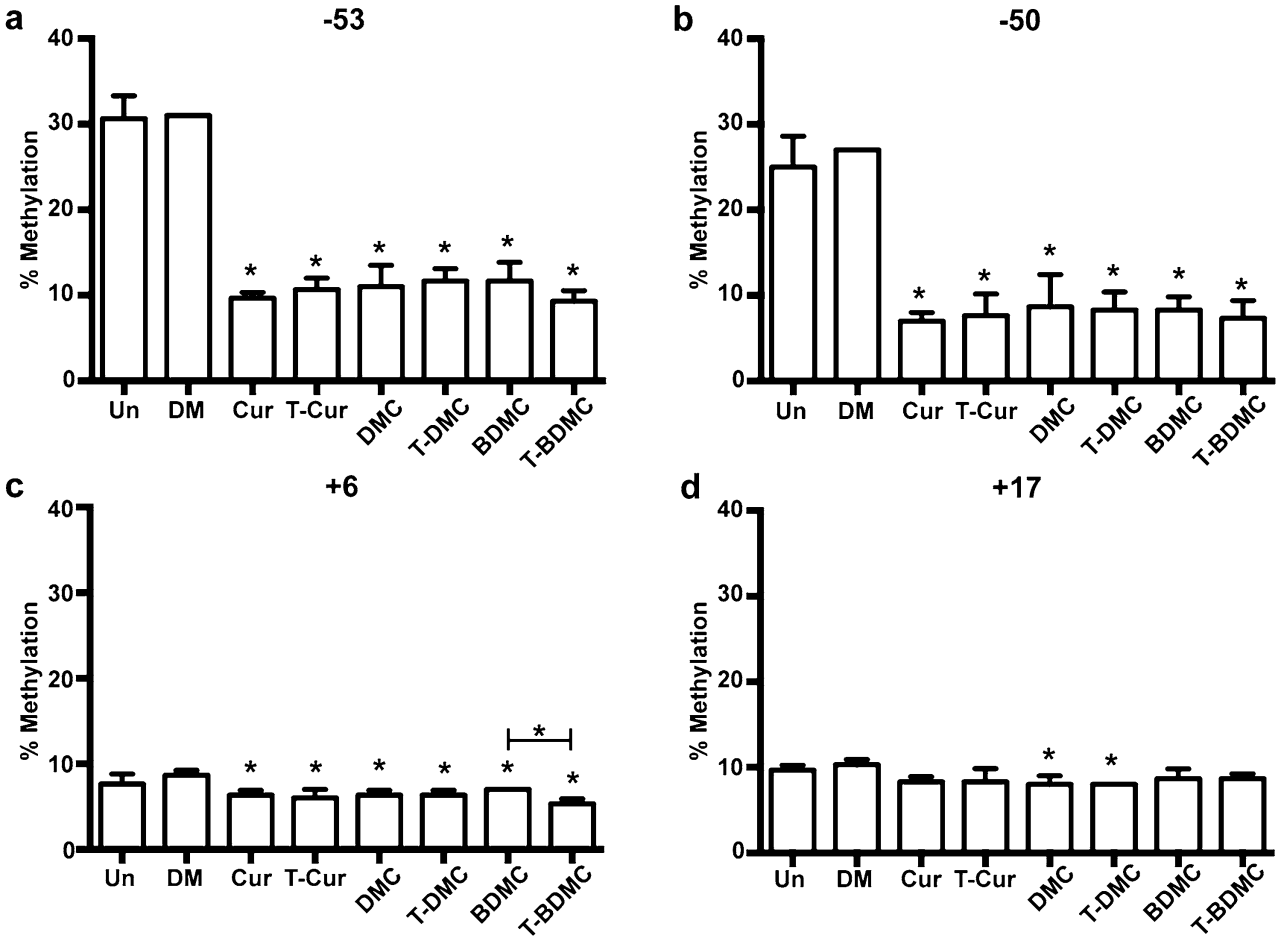
Curcuminoids and their trienone analogs decrease methylation of the ^G^γ-globin promoter in K562 cells. The K562 cells were treated with the compounds for 5 days. DNA methylation at CpG (**a**) − 53, (**b**) − 50, (**c**) + 6 and (**d**) + 17 of the transcription start site were determined by bisulfite conversion and pyrosequencing. The data represents as mean ± SD of three independent experiments. *, statistical significance when compared to DMSO treated cells or between groups at *P* < 0.05.

DNA methylation at γ-globin promoter region was associated with erythroid γ-globin gene switching during ontogenesis. The methylation level and pattern are inversely related to γ-globin gene expression. Fetal stage erythroid cells predominantly expressed γ-globin with hypomethylation pattern while hypermethylation pattern was found in adult stage erythroid cells with low γ-globin expression^11^. To further assess whether HbF synthesis induced by the trienone compounds associated with DNA methylation, methylation status of the 4 CpG sites; − 53, − 50, + 6 and + 17 in both ^G^γ-globin and ^A^γ-globin promoters were determined by bisulfite conversion and pyrosequencing. At CpG − 53 and − 50 of ^G^γ-globin promoter, DNA methylation of K562 cells treated with all six compounds was reduced to approximately 10%. Additionally, DNA methylation at CpG + 6 of ^G^γ-globin promoter was decreased about 1–3% (Fig. 3). DNA methylation of K562 cells treated with T-BDMC at CpG − 53, − 50 and + 6 (9.3 ± 1.7%, 7.3 ± 1.7% and 5.3 ± 0.5%, respectively) was significantly lower than that of control cells (30.7 ± 3.8%, 25.0 ± 2.9% and 7.7 ± 0.9%, respectively) (*P* < 0.05). In contrast, the six compounds have no effect on DNA methylation at the four CpG of ^A^γ-globin promoter region (Supplementary Fig. 1). It is noteworthy that DNA methylation of untreated cells at CpG − 53 and –50 of ^G^γ-globin (30.7 ± 3.8 and 25.0 ± 2.9, respectively) was significantly higher than ^A^γ-globin (4.0 ± 0.8 and 2.7 ± 0.5, respectively) (*P* < 0.05).

### Trienone compounds induce HbF synthesis in β-thalassemia/HbE erythroid progenitors

**Figure 4:**
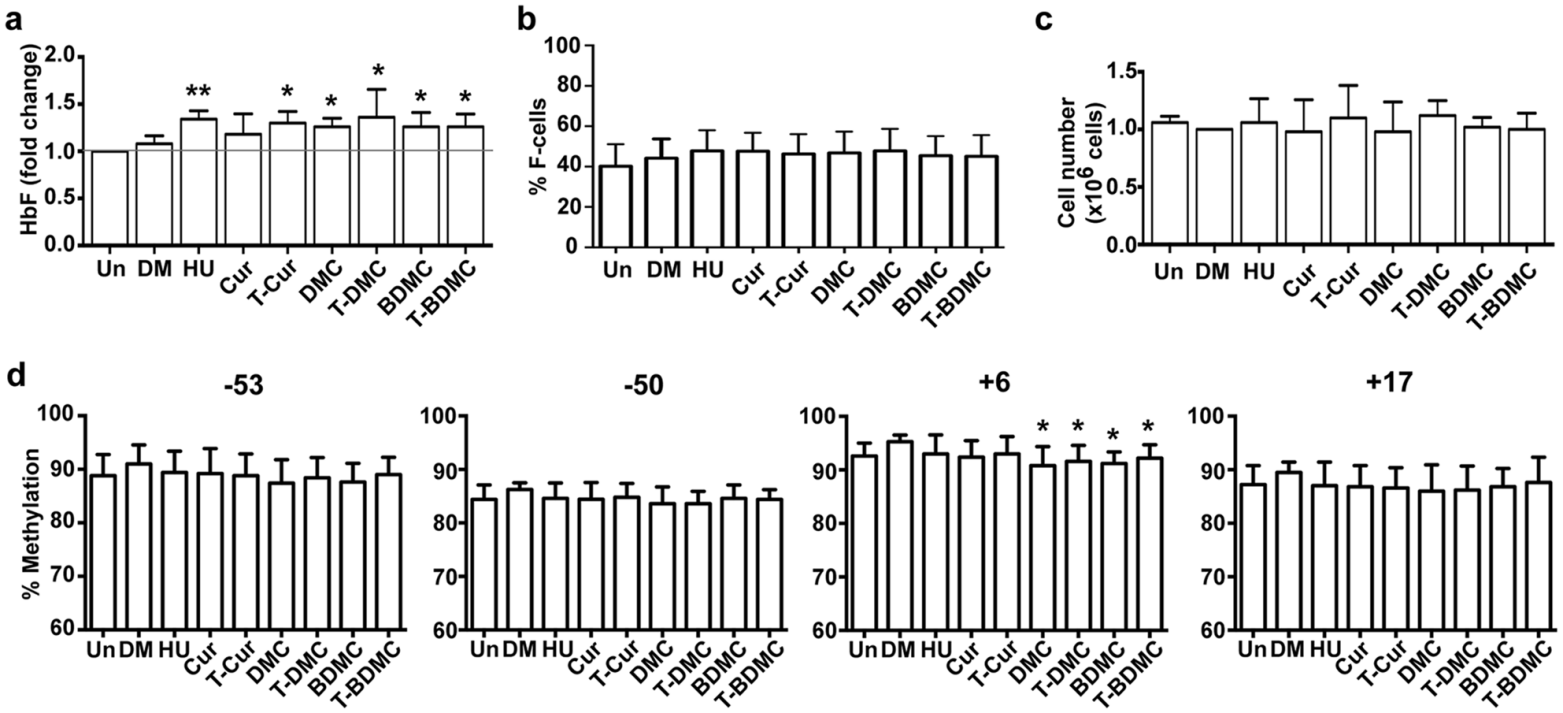
Curcuminoids and their trienone analogs induce HbF synthesis in β-thalassemia/HbE erythroid progenitor cells. The erythroid precursor cells were treated on day 7 of culture and harvested on day 11 for determination of HbF by immunostaining with FITC-conjugated anti-HbF and analyzed by flow cytometry. Analysis of fold change of (**a**) HbF, (**b**) % F-cells and (**c**) cell number of β-thalassemia/HbE erythroid progenitors were compared to untreated cells. Increased HbF in the compounds treated cells compared with DMSO treated cells was observed without significant reduction in cell number compared with untreated cells. (**d**) DNA methylation at CpG − 53, − 50, + 6 and + 17 of the ^G^γ-globin promoter region were determined by bisulfite conversion and pyrosequencing. Decreases DNA methylation at CpG + 6 in treated cells was observed. HU (100 μM) was used as positive control. DMSO (0.25%) was used as negative control. The data were shown as mean ± SD from five β-thalassemia/HbE patients (n = 5). *,**, statistical significance when compared to DMSO treated cells at *P* < 0.05 and *P* < 0.01, respectively.

The HbF inducing property of the trienone compounds was further validated in β-thalassemia/HbE erythroid progenitors. As expected, the thalassemic primary erythroid progenitor cells treated with 100 μM HU (1.3 ± 0.1 fold) was significantly induced HbF synthesis compared to DMSO treated cells, (1.1 ± 0.1-fold) (*P* < *0.01*). All curcuminoids and trienone compounds, except Cur significantly increased HbF synthesis in β-thalassemia/HbE erythroid progenitors (Fig. 4a). However, unlike K562 cells, there was no significant difference in HbF induction effect among the five compounds in β-thalassemia/HbE erythroid progenitors. The % F-cells of β-thalassemia/HbE erythroid progenitors treated with the compounds were slightly increased compared to untreated cells but not reach statistical significance (Fig. 4b). In addition, there was no reduction in cell number of curcuminoids and trienones treated cells compared with untreated cells (Fig. 4c).

### Trienone compounds decreased DNA methylation at γ-globin promoter of β-thalassemia/HbE erythroid progenitors

DNA methylation at the four CpG of γ-globin promoter region in β-thalassemia/HbE erythroid progenitors was examined in order to determine whether HbF induction is associated with DNA methylation as suggested by the results of K562 cell. DNA methylation at + 6 of ^G^γ-globin was significantly decreased in β-thalassemia/HbE erythroid progenitors treated with DMC (90.8 ± 3.2%), BDMC (91.2 ± 1.2%), T-DMC (91.6 ± 2.7%) and T-BDMC (92.2 ± 2.2%) compared to the DMSO treated cells (95.3 ± 1.1%) (*P* < *0.05*) (Fig. 4d). However, DNA methylation at CpG position − 53, − 50 and + 17 was not decreased after treatment with all curcuminoids and trienone compounds. Similarly to K562 cells, the six curcuminoids and trienone compounds had no effect to DNA methylation at the four CpG of ^A^γ-globin (Supplementary Fig. 2).

## Discussion

The imbalance of globin chain synthesis is a major cause of clinical severity and pathophysiology in β-thalassemia. Augmentation of HbF synthesis is an alternative treatment which can improve the imbalance of globin chains synthesis and severity of β-thalassemia^1^. Several HbF inducing agents still are low efficacy, less specificity and toxicity concerned. The only US FDA-approved drug for β-hemoglobinopathies treatment, HU, has side effects, cytopenia and bone marrow suppression^17^. In addition, it exhibits variable responder magnitudes. Only roughly half of transfusion dependent β-thalassemia patients are responders with no need of blood transfusion after using HU. On the other hand, about 20% are non-responders who remain transfusion dependent and the rest are partial responders who have increased hemoglobin levels but still need transfusions^7, 8^. Therefore, many researches have put an effort to find new HbF enhancers with great efficiency and less toxicity. Several plant constituents have been reported to induce HbF synthesis such as resveratrol from grapes^18^, bergapten from *Citrus bergamia*^19^, angelicin from *Aegle marmelos*^20^, quercetin from *Anaxagorea luzonensis*^21^, labdane diterpenes from the aerial parts of *Curcuma comosa*^22^ and curcuminoids from *C. longa*^9^. Here we showed that rare natural curcuminoid analogs, T-Cur and T-BDMC, and the synthetized trienone analogs, T-DMC, have HbF inducing property in both K562 and β-thalassemia/HbE erythroid progenitor cells.

Structure–activity relationship study indicated that the presence of heptatrienone moiety in the curcuminoid analogs resulted in higher effect on HbF induction than that of α,β-unsaturated β-diketo moiety of natural curcuminoids as the trienone analogs have higher HbF induction activity when compared to their corresponding curcuminoids in K562 cells. In addition, the absence of *meta*-methoxy groups at the two aromatic rings also increased the efficiency of HbF synthesis. Among trienone analogs and curcuminoids, T-BDMC and BDMC appeared to have the highest EGFP expression and HbF synthesis inducing activity in K562 cells. These results are consistent with the previous study, which demonstrated that BDMC gave a higher EGFP expression and HbF synthesis compared to other curcuminoids^9^.

HbF inducing agents act through various mechanisms. HU has more effect in increasing F-cells. A combination of HU with a second HbF inducer increased more HbF expression in sickle cell disease patients as the second HbF inducer produce higher concentrations of HbF content in erythroid cells primed by HU, which in turn contribute to higher total HbF^23^. Although a higher % F-cells and HbF expression per cells is anticipate in a longer treatment than 4 days reported here. Our results showed that the trienone analogs of curcuminoids could induce HbF in β-thalassemia/HbE erythroid progenitors. Though the trienone analogs of curcuminoids did not increased % F cells, combination with HU may also leading to additive or synergistic HbF inducing effect.

β-Thalassemia causes by hundreds of mutations with various molecular mechanisms. In addition, there are multiple genetic modifiers associated with β-thalassemia disease severity and HbF expression^24–26^. The variation in individual response to HU attributed to several factors including different thalassemia mutations, α globin chain production and variable interactions of genetic modifiers. Correlation between response to HU and α-thalassemia and β-thalassemia mutations has also been reported^27, 28^. HbF-quantitative trait loci (QTL) such as the *Xmn*I polymorphism and *BCL11A* has been showed to be associated with HU responsiveness^27, 29^. As genetic makeup of the patients determine HbF inducer response, a single candidate compound is not likely to ameliorate all the wide range of genetic variation in the patients. Therefore, a new compound or combination of compounds might be used to achieve therapeutic HbF levels in a wider range of patients.

The exact mechanism whereby curcuminoids increase HbF synthesis remains unclear. DNA methylation is an epigenetic mechanism that controls gene transcription, genome stability and genetic imprinting, including the genes of globin. Two hypomethylating agents, 5-azacytidine and decitabine, could reactivate γ-globin gene expression and HbF synthesis through DNA demethylation in both β-thalassemia and sickle cell disease^2, 3, 30, 31^. However, limitation of long term use of 5-Aza was concerned over tumorigenicity^12^. Decitabine, a safer cytidine analog, still has cytotoxic effect. Curcuminoids, Cur, DMC and BDMC, have been reported as a DNA hypomethylating agent, which inhibit enzymatic activity of DNMT1 by covalently binding with the thiol group of cysteine C1266 at catalytic pocket of DNMT1^14^. In addition, down-regulation of DNMT1 expression by Cur has also been demonstrated^15^. In this study, DNA methylation markedly decreased at CpG position − 53 and − 50 of ^G^γ-globin promoter after treatment with curcuminoids and trienone analogs in K562 cells. The two CpG positions are the location of stage selector element (SSE), which is binding site of transcription factors specificity protein 1 (Sp1) and stage selector protein (SSP), a heterodimer of nuclear erythroid factor 4 (NF-E4) and alpha-globin transcription factor CP2. DNA methylation increases SP1 binding and prevent binding of SSP consequently suppression of γ-globin expression^32^. However, our studies only investigated on DNA methylation modulated by curcuminoids and trienone analogs. Thus, the possibility that the trienone analogs may also modulate on other epigenetic modulators, such as histone acetylation, histone methylation, histone phosphorylation and histone ubiquitination could not be excluded.

The inconsistency in demethylation by the compounds between K562 and β-thalassemia/HbE erythroid progenitor cells might be due to different in nature of cells. K562 cells express mostly embryonic and fetal hemoglobins, while adult β-thalassemia/HbE erythroid progenitor cells express mostly adult hemoglobin. The DNA methylation in β-thalassemia/HbE erythroid progenitors was also increased in K562 cells. Thus, increased HbF synthesis in β-thalassemia/HbE erythroid progenitors might be regulated by other mechanism.

Curcumin is poorly absorbed and undergoes rapid metabolic reduction and conjugation after oral administration resulting in low systemic bioavailability^33, 34^. Curcumin instability and pharmacokinetic deficiency is in part due to the unstable β-diketo moiety. The modification of β-diketo moiety and the methylene group to the mono-keto group improved efficacy, stability and pharmacokinetic properties including bioavailability than that of the α,β-unsaturated β-diketone curcumin analogs^35, 36^. Therefore, the trienone analogs in this study should improve their stability and activity. Recently, the trienone analogs including T-BDMC have been reported as candidates for developing potent anticancer agents against human oral squamous cell carcinoma with less toxicity against Vero cells, a normal cell line, than their respective curcuminoid analogs^10, 37^. However, there is no report about the pharmacokinetic activity and bioavailability of the trienone analogs. Thus, further in vivo study of the compounds is warrant.

In summary, we report for the first time that trienone analogs of curcuminoid increase HbF synthesis in primary human erythroid cell culture. The compounds induced HbF synthesis through demethylation at ^G^γ-globin promoter. In addition to regulation of DNA methylation, further studies are needed to clarify the mechanism of γ-globin inducer of these compounds in order to develop targeted therapeutic strategies for hemoglobin disorders such as β-thalassemia and sickle cell disease.

## Methods

### Natural curcuminoids and trienone compounds

The three natural curcuminoids, Cur, DMC and BDMC, were obtained from the rhizome of *Curcuma longa* as previously described^38^. The trienone compounds; T-Cur, T-DMC and T-BDMC, structurally related to the curcuminoids, were prepared by chemical modification of the corresponding curcuminoids and their structures were confirmed as previously described^10^. The purity of all compounds was determined to be more than 95% by thin-layer chromatography (TLC) and nuclear magnetic resonance (NMR) spectroscopy.

### K562 cell culture and treatment

A stable reporter cell line, K562:Δ^G^γ-^A^γ EGFP cells, harbored enhance green fluorescent protein (EGFP) in replacement of ^G^γ- and ^A^γ-globin coding sequences under the control of ^G^γ-globin promoter intact in human β-globin locus was used as a model for screening of HbF inducers^16^.The reporter cell line and K562 cells were cultured in RPMI1640 media supplemented with 20% fetal bovine serum (FBS; Sigma-Aldrich, St. Louis, MO). The cells were treated with the compounds for 5 days. Cisplatin (20 μM) (Pfizer, Bentley, Australia) and hemin (50 μM) (Sigma-Aldrich) were used as positive controls. DMSO (1%) was used as negative control. Untreated cells were used as analyzed baseline.

### Human erythroid progenitor cell culture and treatment

This study was performed in accordance with the Helsinki declaration and was approved by Mahidol University Institutional Review Board (approval number MU-CIRB 2013/022.1103). Written informed consents were obtained from all individual participants in this study. The total of 5 β-thalassemia/HbE patients were recruited. Mononuclear cells were isolated from peripheral blood by gradient centrifugation with Lymphoprep, density 1.077 g/ml (Axis-Shield, Oslo, Norway). The cells were then cultured in a two-phase culture as previously described^39^. Briefly, the first phase, expansion phase, the mononuclear cells were cultured for 7 days in Iscove’s Modified Dulbecco’s Medium (IMDM, GIBCO-Invitrogen, Carlsbad, CA) containing 30% FBS (Sigma-Aldrich), 25 ng/ml interleukin-3 (IL-3; Cell Signaling Technology, Beverly, MA), 50 ng/ml stem cell factor (SCF; Cell Signaling Technology) and 0.1 U/ml erythropoietin (EPO; Janssen-Cilag, New Brunswick, NJ). The second phase, differentiation phase, progenitor cells from expansion phase were cultured in IMDM supplemented with 30% FBS, 0.1 ng/ml IL-3 and 3 U/ml EPO. On day 7 of the second phase, the erythroid progenitors were treated with curcuminoids and trienone compounds. Cells were harvested on day 11 of the second phase for HbF and mRNA expression and DNA methylation analysis. HU (100 μM) (Bristol-Myers Squibb, Rome, Italy) was used as positive control. DMSO (0.25%) was used as negative control. Untreated cells were used as analyzed baseline control.

### Analysis of EGFP expression and HbF synthesis

The induction of EGFP expression in K562:Δ^G^γ-^A^γ EGFP reporter cells was acquired and analyzed by FACSCalibur flow cytometer and CellQuest Pro software (BD Biosciences, San Diego, CA). For HbF determination, the K562 cells were fixed and permeabilized with fix & perm cell permeabilization kit (GIBCO-Invitrogen) according to the manufacturer and stained with fluorescein isothiocyanate (FITC)-conjugated anti-human HbF monoclonal antibody (BD Biosciences). FITC-conjugated mouse IgG monoclonal antibody (BD Biosciences) was used as an isotype control. The stained K562 cells were acquired and analyzed by FACSCalibur flow cytometer and CellQuest Pro software.

### Cytotoxicity measurement

The viability of K562:Δ^G^γ-^A^γ EGFP reporter cells and K562 cells was measured by staining with 1 μg/ml propidium iodide (PI, Sigma-Aldrich) and then acquired and analyzed by FACSCalibur flow cytometer and CellQuest Pro software. Viable erythroid progenitor cells were enumerated by hematocytometry using trypan blue staining.

### DNA methylation analysis

DNA methylation analysis was performed using bisulfite conversion and pyrosequencing techniques. Bisulfite modification was processed on genomic DNA using the Epitect bisulfite conversion kit (QIAGEN, Valencia, CA) as per the manufacturer's protocol. The ^G^γ- or ^A^γ-globin promoter regions encompass the interested CpG sites at − 53, − 50, + 6 and + 17 were amplified using allele specific semi-nested PCR (Supplementary Fig. 3). The initial round of PCR was performed using forward primers GGF1 (5′-GAAGTTTTGGTATTTTTTATGG-3′) for ^G^γ-globin and AGF1 (5′-GAAGTTTTGGTATTTTTTATGA-3′) for ^A^γ-globin. The semi-nested PCR was performed using forward primers GGF2 (5′-TATTTTTTATGGTGGGAGAAGAA-3′) for ^G^γ-globin and AGF2 (5′-TATTTTTTATGATGGGAGAAGGA-3′) for ^A^γ-globin. The biotinylated reverse primer, GammaR (5′-Biotin-ACTTATAATAATAACCTTATCCTCCTCTA-3′) was used for both initial and nested PCR amplifications of both ^G^γ-globin and ^A^γ-globin. Single-strand biotinylated PCR product was used as template for pyrosequencing using primer 5′-TAGTGAGGTTAGGGG-3′ for CpG positions − 53 and − 50 and primer 5′-AAGTATTTTTAGTAGTTTTATATA-3′ for CpG sites + 6 and + 17. The pyrosequencing was performed using PyroMark Q24 (QIAGEN) and PyroMark CpG SW 1.0 Q24 software (QIAGEN).

### Statistical analysis

Data were reported as mean ± SD and analyzed using SPSS Version 18.0 (IBM Collaboration, Armonk, NY). Comparisons between parameters were evaluated with a non-parametric Mann–Whitney *U* test. The statistical significance of all comparisons was considered at *P* < 0.05.

## Supplementary Information

Supplementary Figures.
