# Supplementary material for: Trienone analogs of curcuminoids induce fetal hemoglobin synthesis via demethylation at Gγ-globin gene promoter
Source: Sci Rep. 2021 Apr 20;11:8552. doi: 10.1038/s41598-021-87738-2 (PMC8058333; doi:10.1038/s41598-021-87738-2)
Supplement: Supplementary file 1 — Supplementary Figures. [file 41598_2021_87738_MOESM1_ESM.pdf]

Supplementary information

Trienone analogs of curcuminoids induce fetal hemoglobin synthesis via demethylation at $^{G}\gamma$-globin gene promoter

Khanita Nuamsee[1,2,3], Thipphawan Chuprajob[4,5], Wachirachai Pabuprapap[4], Pornrutsami Jintaridth[6], Thongperm Munkongdee[2], Phatchariya Phannasil[2], Jim Vadolas[7,8], Pornthip Chaichompoo[3], Apichart Suksamrarn[4], Saovaros Svasti[2,9]

[1]Graduate Program in Molecular Medicine, Faculty of Science, Mahidol University, Bangkok, Thailand. [2]Thalassemia Research Center, Institute of Molecular Biosciences, Mahidol University, Nakhon Pathom, Thailand. [3]Department of Pathobiology, Faculty of Science, Mahidol University, Bangkok, Thailand. [4]Department of Chemistry and Center of Excellence for Innovation in Chemistry, Faculty of Science, Ramkhamhaeng University, Bangkok, Thailand. [5]Department of Chemistry, Faculty of Science, Siam University, Bangkok, Thailand. [6]Department of Tropical Nutrition and Food Science, Faculty of Tropical Medicine, Mahidol University, Bangkok, Thailand. [7]Centre for Cancer Research, Hudson Institute of Medical Research, Melbourne, Australia. [8]Department of Molecular and Translational Science, Monash University, Melbourne, Australia. [9]Department of Biochemistry, Faculty of Science, Mahidol University, Bangkok, Thailand.

Number of supplementary figure: 3

Contact information for Corresponding Author

Saovaros Svasti, Ph.D.

Thalassemia Research Center, Institute of Molecular Biosciences, Mahidol University

25/25 Phuttamonthon 4 Road, Salaya, Nakhon Pathom 73170 Thailand.

Tel: +66-2889-2557, Fax: +66-2889-2559

E-mail: saovaros.sva@mahidol.ac.th, stssv@yahoo.com

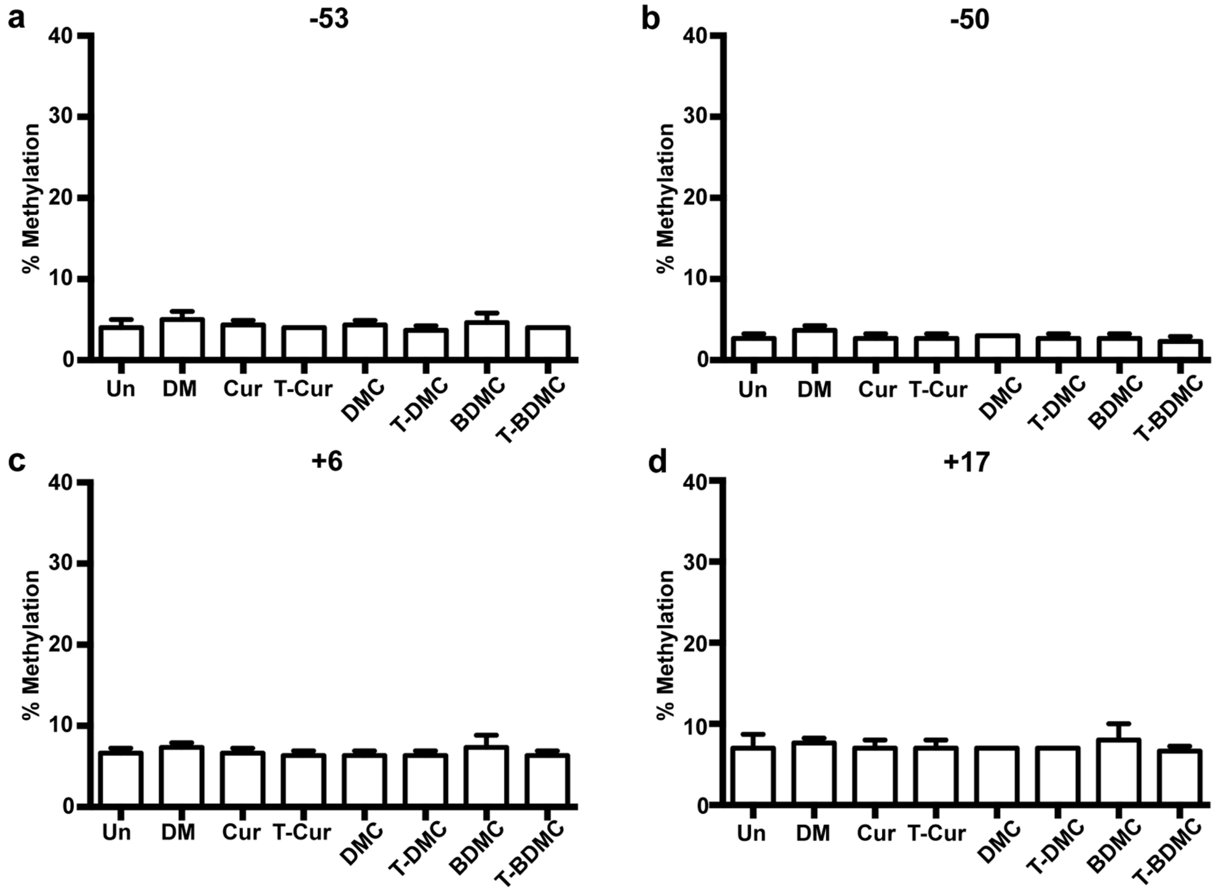

Supplementary Fig. 1 DNA methylation at $^{A}\gamma$-globin promoter region of K562 cells treated with curcuminoids and their trienone analogs. The K562 cells were treated with the compounds for 5 days. DNA methylation at CpG (A) -53, (B) -50, (C) +6 and (D) +17 of the transcription start site were determined by bisulfite conversion and pyrosequencing. The data represents as mean±SD of three independent experiments.

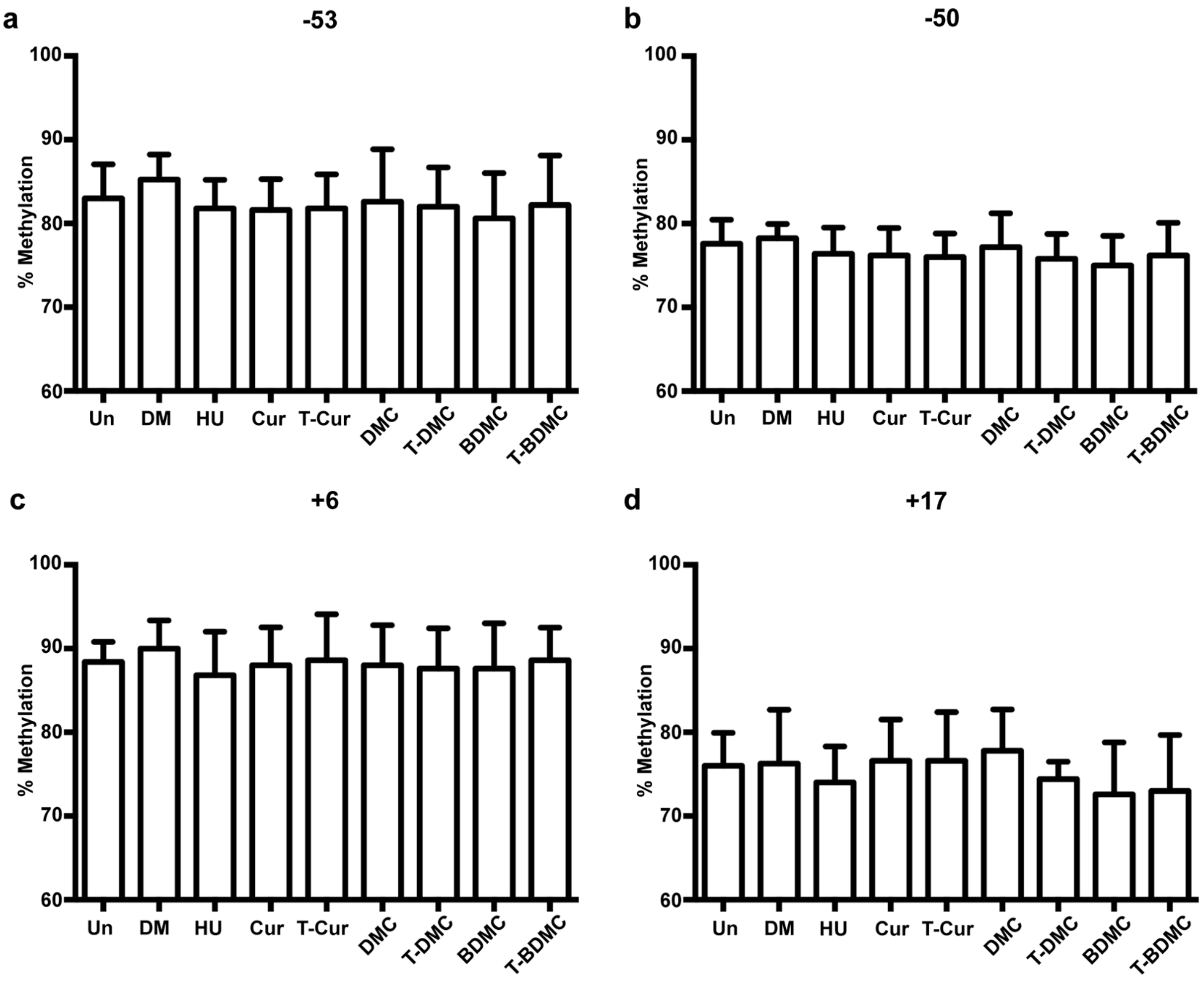

Supplementary Fig. 2 DNA methylation at $^{A}\gamma$-globin promoter region of β-thalassemia/HbE erythroid progenitor cells treated with curcuminoids and their trienone analogs. DNA methylation at CpG (A) -53, (B) -50, (C) +6 and (D) +17 of $^{A}\gamma$-globin promoter region were determined by bisulfite conversion and pyrosequencing. The data were shown as mean±SD from five β-thalassemia/HbE patients (n = 5).

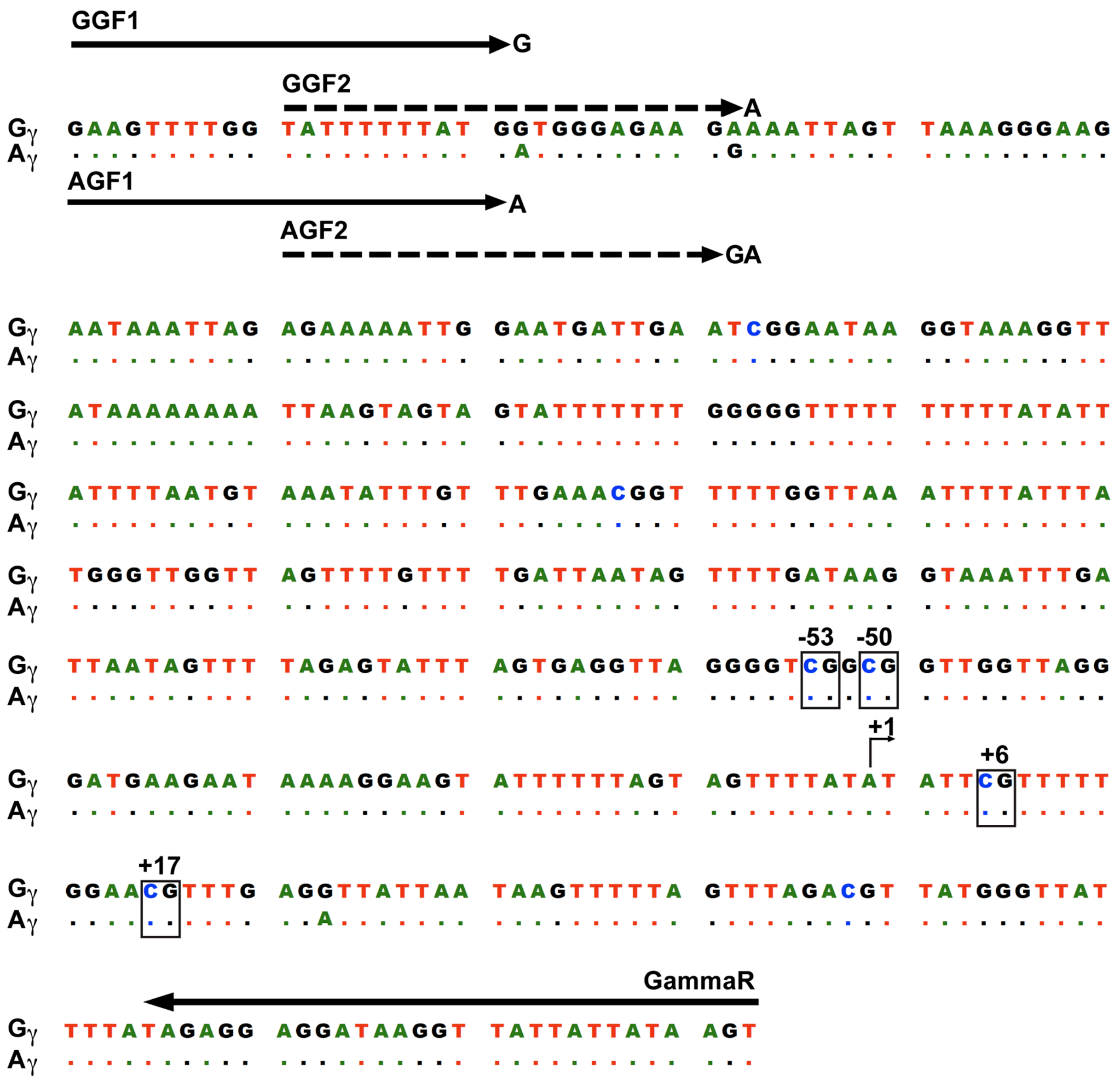

Supplementary Fig. 3 Sequence alignment of $^{G}\gamma$-globin and $^{A}\gamma$-globin promoter regions and the positions of PCR primers. The GGF and AGF represent the forward primers that specifically amplify to $^{G}\gamma$-globin and $^{A}\gamma$-globin gene promoter, respectively. The GammaR represents the 5′-end biotinylated reverse primer specific to both $^{G}\gamma$-globin and $^{A}\gamma$-globin gene promoter. Arrows represent the direction of PCR primers. Transcription start site (+1), position of the four CpG positions is indicated in the square box.
